# Compilation of Genotype and Phenotype Data in *GCDH*-LOVD for Variant Classification and Further Application

**DOI:** 10.3390/genes14122218

**Published:** 2023-12-14

**Authors:** Alexandra Tibelius, Christina Evers, Sabrina Oeser, Isabelle Rinke, Anna Jauch, Katrin Hinderhofer

**Affiliations:** Institute of Human Genetics, Heidelberg University, 69120 Heidelberg, Germany

**Keywords:** glutaric aciduria, glutaric acidemia, glutaryl-CoA dehydrogenase, *GCDH*, inborn errors of metabolism, variant interpretation, variant classification, geographic distribution, LOVD, variation database

## Abstract

Glutaric aciduria type 1 (GA-1) is a rare but treatable autosomal-recessive neurometabolic disorder of lysin metabolism caused by biallelic pathogenic variants in glutaryl-CoA dehydrogenase gene (*GCDH*) that lead to deficiency of GCDH protein. Without treatment, this enzyme defect causes a neurological phenotype characterized by movement disorder and cognitive impairment. Based on a comprehensive literature search, we established a large dataset of *GCDH* variants using the Leiden Open Variation Database (LOVD) to summarize the known genotypes and the clinical and biochemical phenotypes associated with GA-1. With these data, we developed a *GCDH*-specific variation classification framework based on American College of Medical Genetics and Genomics and the Association for Molecular Pathology guidelines. We used this framework to reclassify published variants and to describe their geographic distribution, both of which have practical implications for the molecular genetic diagnosis of GA-1. The freely available *GCDH*-specific LOVD dataset provides a basis for diagnostic laboratories and researchers to further optimize their knowledge and molecular diagnosis of this rare disease.

## 1. Introduction

Glutaric aciduria type 1 (GA-1) is a rare autosomal-recessive neurometabolic disorder with a worldwide incidence of 1:100,000 [1]. Deficiency of the mitochondrial enzyme glutaryl-CoA dehydrogenase (GCDH) leads to accumulation of the neurotoxic glutaric acid (GA) and 3-hydroxyglutaric acid (3-OH-GA). The phenotypic spectrum of untreated GA-1 ranges from severe acute encephalopathy with striatal degeneration, dystonia, and chorea in the first year of life to a rare adult-onset form with non-specific neurological symptoms [2,3,4]. Early metabolic treatment can prevent irreversible neurological disease due to striatal injury, so GA-1 has been included in newborn screening (NBS) in many countries [5,6,7,8]. According to recent guidelines for the diagnosis and management of GA-1, patients with a positive NBS or a suspected clinical diagnosis of GA-1 should undergo biochemical analysis with quantitative measurement of GA and 3-OH-GA in the blood and/or urine. If 3-OH-GA is elevated, treatment should be administered immediately. A GA-1 diagnosis can then be confirmed by molecular genetic analysis of the *GCDH* gene. However, a correlation between genotype and clinical course of the disease has not been established so far [3,5,9,10,11,12]. If the patient does not want genetic analysis or if the analysis fails to detect two biallelic pathogenic *GCDH* variants, GCDH enzyme activity in leukocytes or fibroblasts can be analyzed to confirm the diagnosis [13].

Genetic sequencing of the coding regions and exon–intron boundaries of *GCDH* has high sensitivity [14]. The identification of two biallelic pathogenic or likely pathogenic *GCDH* variants is not only relevant for diagnosis but also for genetic counseling of patients and their families, such as carrier testing in relatives. Prenatal diagnosis or preimplantation genetic diagnosis is feasible if pathogenic *GCDH* variants have been identified in the index patient. The cost of next-generation sequencing (NGS) is declining and its availability in medical diagnostics is increasing rapidly worldwide; therefore, NGS will likely replace biochemical analysis as the first diagnostic step after a positive NBS result [15].

In 2015, the American College of Medical Genetics and Genomics (ACMG) and the Association for Molecular Pathology (AMP) described a framework for more reproducible interpretation of sequence variants according to several criteria with different levels of evidence [16]. These widely used ACMG–AMP recommendations have been modified and adapted by ClinGen Working Groups (www.clinicalgenome.org; accessed on 15 September 2023) for different diseases/genes, such as the phenylalanine hydroxylase (*PAH*) gene [17]. In addition, the Association for Clinical Genomic Science (ACGS) has published modified guidelines for classifying variants in patients with rare monogenetic diseases with high penetrance [18].

Genetic variant databases are an essential tool for interpreting variants. One such database is the Leiden Open Variation Database (LOVD), a free available open-source database of genomic variants [19]. To maintain high-quality data, gene experts (curators) revise submitted information on patients and variants. In contrast to other genetic databases, LOVD compiles information not only on various genetic variants but also on patient genotypes and phenotypes. Information on genotypes is especially important for interpreting variants in autosomal-recessive diseases. Further, information on phenotypes is helpful when interpreting variants in rare disorders, especially when exome or genome-wide sequencing is used for diagnostic testing. Another advantage of LOVD is that additional information can be submitted, such as family history, geographic origin, patient treatment, disease course, and functional analysis of variants.

We performed a comprehensive literature search and submitted the extracted data to LOVD. The aim of this work was to merge all GA-1 patients published to date into a single large dataset within LOVD that would summarize all known genotypes and phenotypes associated with this rare disease. The dataset was used (a) to reevaluate published *GCDH* variants to develop a strategy for variant classification according to the current guidelines and recommendations, and (b) to investigate the geographic distribution of the variants. We modified existing rules to assess the pathogenicity of rare genetic variants using the LOVD database infrastructure, and provided another example for variant classification in rare diseases. This modified GA-1 variant classification system is not intended to be a new guideline for *GCDH* variant interpretation, but rather an approach to interpreting *GCDH* variants. This system is still being developed and can be further optimized for clinical use.

## 2. Materials and Methods

### 2.1. Literature Search, Submission to LOVD, and Systematic Compilation of GA-1 Genotypes and Phenotypes

A systematic literature search was conducted on PubMed for articles published up to August 2023 on *GCDH* variants in GA-1 patients. For this search, the following MeSH terms were used: “glutaric aciduria type 1,” “glutaric acidemia type 1,” “glutaryl-CoA dehydrogenase deficiency,” and “*GCDH*.” Existing information on the *GCDH* locus in LOVD was enhanced with available genotypes and phenotypes of patients from the literature. A total of 842 individuals and 306 different variants were found for further analysis.

### 2.2. Variant Evaluation

A total of 306 unique variants in the long isoform of *GCDH* (RefSeq NM_000159.2) were evaluated. For each variant, LOVD individuals in which the variant has been reported were counted. Information on genotypes of corresponding individuals was used to report the zygosity, the pathogenicity of the co-occurring variant, and the phase of variants. If available, evidence from functional studies was added.

Variants were analyzed in silico with the clinical prediction software Alamut Visual Plus version 1.6.1 (SOPHiA GENETICS SA, Saint-Sulpice, Switzerland) comprising multiple variant database information and in silico tools, including meta-predictor REVEL [20,21]. Potential effects on splicing were examined with splicing prediction apps SpliceVault (https://kidsneuro.shinyapps.io/splicevault/; accessed on 29 September 2023) [22] and SpliceAI (https://spliceailookup.broadinstitute.org/; accessed on 29 September 2023) [23]. Variant frequencies and missense constraint metrics were obtained from gnomAD v2.1.1 (https://gnomad.broadinstitute.org; accessed on 29 September 2023) and DECIPHER (https://www.deciphergenomics.org/; accessed on 29 September 2023).

### 2.3. Variant Classification

ACMG/AMP rules were applied with modifications according to current recommendations of the ClinGen Sequence Variant Interpretation (SVI) Working Group and ACGS Best Practice Guidelines 2020. See Table 1 for a summary of ACMG/AMP criteria used in this study. Obtained types and levels of evidence were combined for variant classification utilizing the Bayesian point system [24,25]. This system enables a further differentiation of variants classified as variants of uncertain significance (VUS) depending on different levels of their uncertainty.

*GCDH*-specific adjustments of ACMG/AMP criteria concerned allele frequency thresholds, definition of phenotype specific criteria, and application of data from functional studies.

#### 2.3.1. Population and Allele Frequency Data (PM2, BA1, BS1)

The BA1 threshold was set at 0.283% or 0.00283 based on maximum credible population allele frequency determined by using an allele frequency calculator (https://cardiodb.org/allelefrequencyapp/; accessed on 29 September 2023) [26]. The following input parameters were included in the calculation: biallelic inheritance, disease incidence of 1 in 100,000 [1], allelic heterogeneity of 0.8 (to account for the occurrence of variant c.1204C>T p.(Arg402Trp) in approx. 20% of LOVD individuals), genetic heterogeneity of 1, and penetrance of 0.8 (to account for individuals with late disease onset and for reported rare cases of asymptomatic individuals). The allele frequency of the most recurrent pathogenic *GCDH* variant c.1204C>T p.(Arg402Trp) was used to determine the threshold for BS1 criterion (allele frequency greater than expected for disease). This pathogenic variant has a minor allele frequency of 0.07566% in a European non-Finnish population, so BS1 threshold was set at 0.08% or 0.0008. Deriving from this, PM2 threshold was determined as an order of magnitude lower (0.00008 or 0.008%).

#### 2.3.2. Definition of Phenotype Specificity (PP4)

The examination of the phenotypic spectrum of all individuals in LOVD showed a high diversity in the description of clinical symptoms and biochemical findings. Thus, the PP4 criterion was only applied to *GCDH* variants of individuals whose diagnosis was definitively confirmed by direct analysis of the enzyme activity in fibroblasts or lymphoblasts. If the phasing of variants identified in these individuals was performed, the pathogenic evidence was upgraded to PP4_Moderate.

#### 2.3.3. Functional Data

The predicted missense constraint Z score for *GCDH* is low (0.31 in gnomAD) and no specific structural regions display significant association with missense constraint in the DECIPHER database. Therefore, the use of PP2 (evidence for missense constraint) and PM1 (evidence for mutational hotspot/functional domains without benign variation) criteria are not appropriate for classification of *GCDH* variants.

Several functional studies were reviewed in order to obtain an overview of functional assays used. Multiple investigators performed in vitro GCDH enzyme activity analysis in heterologous cells (*E. coli* or COS-7) [9,11,27]. Furthermore, a considerable number of studies addressed the additional potential impact of missense variants on GCDH function [28,29,30,31,32]. Thus, PS3 was used as supporting evidence from functional studies (PS3_Supporting) if only one study showed a residual mutant enzyme activity of 0–50% measured in vitro in comparison to the wild-type enzyme activity. If a single study performed additional assays and showed an impact on GCDH function (e.g., oligomerization or protein stability), the evidence was upgraded to moderate (PS3_Moderate). If more than one functional study confirmed the deleterious effect of a certain variant on GCDH function, PS3 was applied as strong evidence.

Data from RNA analysis showing an impact on splicing were integrated into the application of the PVS1 criterion as recommended by SVI Splicing Subgroup recommendations [33].

#### 2.3.4. Use of PS4

Given the rarity of GA-1, there are no case–control studies that can be used for the calculation of odds ratios and levels of significance for application of PS4. Under these circumstances, ACGS Best Practice Guidelines 2020 advise using PS4 *as a moderate level of evidence if the variant has been previously identified in multiple (two or more) unrelated affected individuals), or as supporting level of evidence if previously identified in one unrelated affected individual, and has not been reported in gnomAD* [18]. Thus, the number of LOVD individuals bearing the variant of interest was factored in for use of the PS4 criterion. Of note, only if the variant was linked to two individuals in the database could PS4_Supporting be applied in line with the specification *previously reported in one unrelated affected individual*.

**Table 1:** ACMG/AMP criteria adapted and specified for *GCDH* variants in LOVD.

Pathogenic Criteria		
Criteria	Criteria Description	Specifications
Very strong criteria		
PVS1	Null variant: nonsense or frameshift	Subject to SVI recommendations [34]
	Null variant: canonical ±1,2 splice variants	Subject to SVI Splicing Subgroup recommendations [33]
PM3_Very Strong	Detected in trans with a pathogenic or likely pathogenic variant ≥4 points	Subject to SVI recommendations [35] Point-based system (e.g., phase confirmed: detected in three compound heterozygotes with three different variants classified as pathogenic or likely pathogenic *and* two homozygotes)
**Strong criteria**		
PVS1_Strong	Null variant: nonsense or frameshift	Subject to SVI recommendations [34]
	Null variant: canonical ±1,2 splice variants	Subject to SVI Splicing Subgroup recommendations [33]
PM3_Strong	Detected in trans with a pathogenic or likely pathogenic variant ≥2 points < 4 points	Subject to SVI recommendations [35] Point-based system (e.g., phase confirmed: detected in two compound heterozygotes with two different variants classified as pathogenic or likely pathogenic)
PS1	Same amino acid change as a previously established pathogenic variant regardless of nucleotide change	None
	Same predicted splicing event as a known pathogenic or likely pathogenic variant	Subject to SVI Splicing Subgroup recommendations [33]
PS3	Well-established in vitro functional studies demonstrating a damaging effect Multiple functional studies show an impact on GCDH function *and* at least one study shows a residual enzyme activity of 0–50%	Adjusted to GA-1
PP3_Strong	Computational (in silico) tools predict a deleterious effect Missense variants: REVEL score ≥ 0.932	Subject to SVI recommendations [36]
**Moderate criteria**		
PVS1_Moderate	Null variant: nonsense or frameshift	Subject to SVI recommendations [34]
	Null variant: canonical ±1,2 splice variants	Subject to SVI Splicing Subgroup recommendations [33]
	Null variant: initiation codon	Subject to SVI recommendations [34]
PM3	Detected in trans with a pathogenic or likely pathogenic variant ≥1 point < 2 points	Subject to SVI recommendations [35] Point-based system (e.g., phase not confirmed: detected in two probands with two different variants classified as pathogenic *or* phase confirmed: detected in one compound heterozygote with variant classified as pathogenic or likely pathogenic)
PS1_Moderate	Same predicted splicing event as a pathogenic or likely pathogenic variant	Subject to SVI Splicing Subgroup recommendations [33]
PS4_Moderate	The prevalence of the variant in affected individuals is significantly increased compared with the prevalence in controls Variant has been previously identified in >2 unrelated affected individuals	Subject to ACGS 2020 guidelines [18]
PS3_Moderate	Well-established in vitro functional studies demonstrating a damaging effect Single functional study shows more than one effect on GCDH function (e.g., residual enzyme activity of 0–50% *and* oligomerization *or* interactions *or* cellular GCDH levels)	Adjusted to GA-1
PP3_Moderate	Computational (in silico) tools predict a deleterious effect Missense variants: REVEL score [0.773, 0.932)	Subject to SVI recommendations [36]
PM4	Protein length changes as a result of in-frame deletions/insertions in a non-repeat region or stop-loss variants In-frame indels greater than a single amino acid	Subject to ACGS 2020 guidelines [18]
PM5	Missense change at amino acid residue where a different missense change determined to be pathogenic or likely pathogenic has been seen before likely pathogenic variant has been identified in more than one case	Subject to ACGS 2020 guidelines [18]
PP4_Moderate	Residual GCDH activity measured in patient cells < 50% *and* variants confirmed in trans	Adjusted to GA-1
**Supporting criteria**		
PM2_Supporting	Absent from controls, or at extremely low frequency Allele frequency threshold < 0.0008 (0.008%)	Adjusted to GA-1 Strength is subject to SVI recommendations [37]
PM3_Supporting	Detected in trans with a pathogenic or likely pathogenic variant ≥0.5 point < 1 point	Subject to SVI recommendations [35] Point-based system (e.g., phase not confirmed → detected in two probands with two different variants classified as likely pathogenic *or* in one proband with variant classified as pathogenic)
PS1_Supporting	Same predicted splicing event as a known pathogenic or likely pathogenic variant	Subject to SVI Splicing Subgroup recommendations [33]
PS4_Supporting	The prevalence of the variant in affected individuals is significantly increased compared with the prevalence in controls Variant has been previously identified in 1 unrelated affected individual *and* PM2 is fulfilled	Subject to ACGS 2020 guidelines [18]
PS3_Supporting	Well-established in vitro functional studies demonstrating a damaging effect Single functional study performed and shows only one effect on GCDH function (e.g., residual enzyme activity of 0–50% *or* oligomerization *or* interactions *or* cellular GCDH levels)	Adjusted to GA-1
PP3_Moderate	Computational (in silico) tools predict a deleterious effect	
	Missense variants: REVEL score [0.644, 0.773)	Subject to SVI recommendations [36]
	Splice site variants outside the canonical splice acceptor and donor regions *and* synonymous variants: SpliceAI Δ score ≥ 0.2	Subject to SVI Splicing Subgroup recommendations [33]
PM4_Supporting	Protein length changes as a result of in-frame deletions/insertions in a non-repeat region or stop-loss variants In-frame indels of a single amino acid	Subject to ACGS 2020 guidelines [18]
PM5_Supporting	Missense change at amino acid residue where a different missense change determined to be pathogenic or likely pathogenic has been seen before likely pathogenic variant has been identified in one case	Subject to ACGS 2020 guidelines [18]
PP4	Residual GCDH activity measured in patient cells < 50% *and* phase of variants unknown	Adjusted to GA-1
**Benign criteria**		
**Stand-alone criteria**		
BA1	Allele frequency above 0.00283 (0.283%)	Adjusted to GA-1
**Strong criteria**		
BS1	Allele frequency greater than expected for disease (0.0008–0.00316 or 0.08–0.316%)	Adjusted to GA-1
**Supporting criteria**		
BP4	Computational (in silico) tools predict no impact on gene or gene product	
	Missense variants: REVEL score [0.183, 0.290]	Subject to SVI recommendations [36]
	Splice site variants outside the canonical splice acceptor and donor regions *and* synonymous variants: SpliceAI Δ score ≤ 0.1	Subject to SVI Splicing Subgroup recommendations [33]
BP7	Synonymous (silent) variants and intronic variants outside donor and acceptor splice regions *and* BP4 fulfilled	Subject to SVI Splicing Subgroup recommendations [33]

### 2.4. Assessment of Variant Distribution across Different Geographic Regions

To determine the geographic distribution of variants, all available information on geographic background was extracted from the above mentioned updated LOVD dataset.

Eleven geographic regions were identified and defined as follows: “Africa” comprised data from Egypt, Morocco, and South Africa; “Central Europe” included data from Austria, Belgium, Czech Republic, Denmark, Germany, the Netherlands, Poland, Slovakia, Slovenia, and Switzerland; “North and Central America” included data from Canada, Mexico, and the United States of America; “South America” comprised data from Brazil, Chile, and Venezuela; “Southeast and East Asia” included data from China, Japan, Malaysia, Taiwan/the Republic of China, and the Republic of Korea; “Southern and Eastern Europe” comprised data from Croatia, Cyprus, Greece, Italy, Portugal, Romania, and Spain; “Turkey and Near East” included data from Iran, Iraq, Israel, Jordan, Saudi Arabia, Syria, and Turkey; “UK and Ireland” comprised data from the United Kingdom and Ireland; and “Australia,” “India” and “Russia” comprised data from the respective regions only.

## 3. Results

### 3.1. Review of GCDH Variants

**Figure 1:**
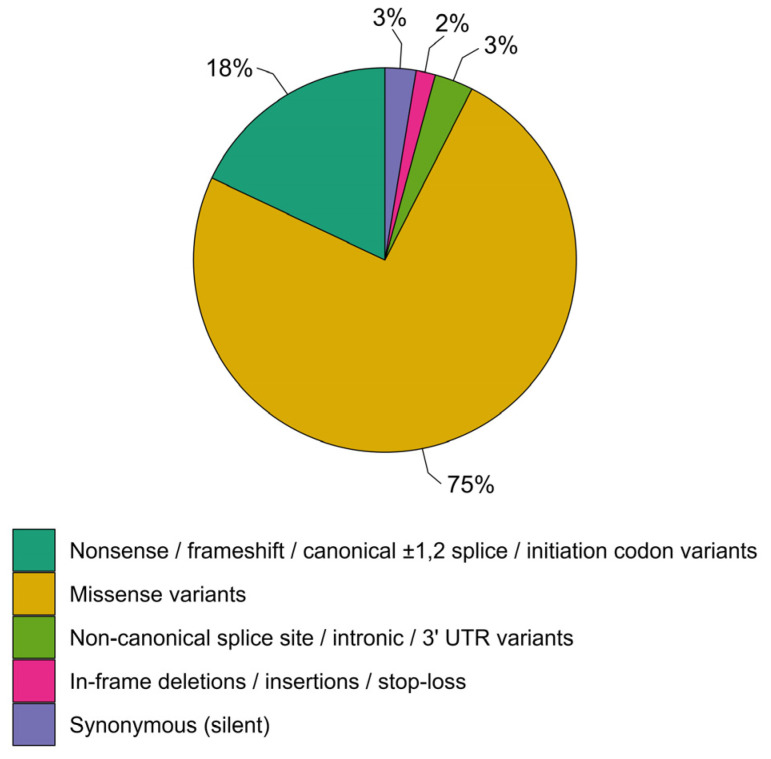
Spectrum of the *GCDH* variants compiled in LOVD.

To date, 306 unique *GCDH* variants have been gathered in LOVD. The spectrum of *GCDH* variants is clearly dominated by missense variants (228/306, 75%), with nonsense, frameshift, canonical ±1,2 splice sites, and initiation codon variants making up a relatively small proportion (55/306, 18%) (Figure 1). In-frame indels, stop loss variants, and variants in non-canonical splice sites, introns, and the 3′ UTR are quite rare (4/306, 1/306, 6/306, 1/306, and 3/306, respectively). Of note, only a few synonymous variants are present in LOVD (*n* = 8). However, this small fraction does not reflect the true abundance of this type of *GCDH* variant as presented in gnomAD.

**Figure 2:**
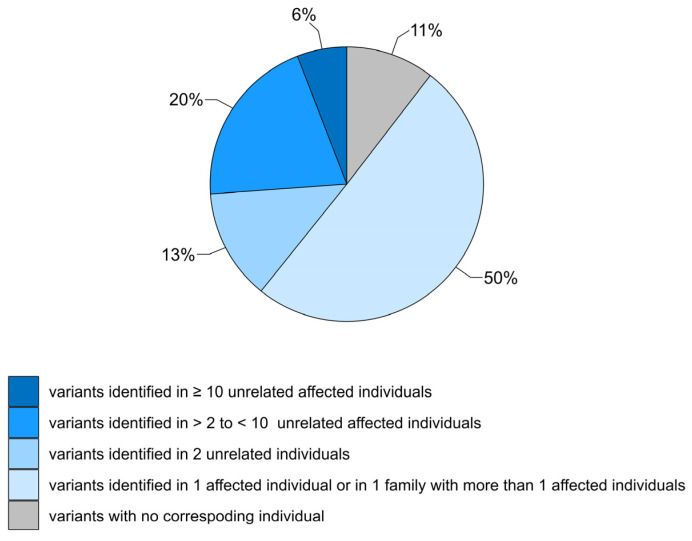
Overview of *GCDH* variant frequencies in LOVD individuals.

Patient data, including the genotype and phenotype, were available for 274 variants. No patient data were provided for the remaining 32 variants, some of which were submitted to the database by users (Figure 2). Half of the variants (154/306, 50%) were observed in a single patient or in a single family with two affected relatives. About a third of these rare variants (48/154) were homozygous, and homozygosity was confirmed by parental testing in 17 cases. With the exception of sporadic cases in which just one heterozygous variant was detected in the affected individual, most rare variants were compound heterozygous with a pathogenic or likely pathogenic *GCDH* variant; however, unambiguous evidence for in trans occurrence was provided for 14 variants only.

Residual GCDH activity measurements in fibroblasts were reported for 161 individuals submitted to LOVD. Several of these individuals had the same genotype (p.[Arg402Trp]; [Arg402Trp] in 14 individuals and p.[Ala293Thr]; [Ala293Thr] in 11 individuals), so GCDH activity was only assessed for 93 different variants. The genotype was confirmed by phasing of variants in 19 of 161 individuals. With the exception of two siblings, 17 individuals had different, mainly compound heterozygous, genotypes.

Functional studies were reported for 56 variants, 22 of which were investigated by different groups. The most common GA-1-associated variants p.(Arg402Trp), p.(Ala421Val), p.(Val400Met), p.(Pro248Leu), p.(Arg227Pro), and p.(Glu365Lys) were often included in the studies.

### 3.2. Summary of Variant Classification Using ACMG/AMP Variant Interpretation Criteria Adjusted for GCDH

**Figure 3:**
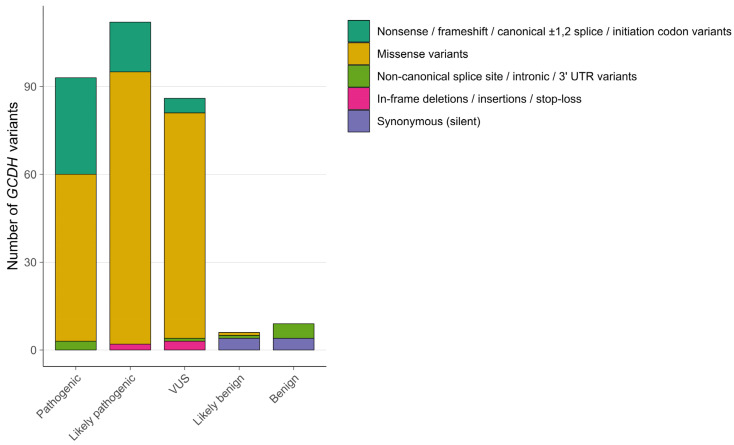
Classification of *GCDH* variants compiled in LOVD.

From the 306 variants evaluated in this study, 93 were classified as pathogenic, 112 as likely pathogenic, 86 as VUS, six as likely benign, and nine as benign (Figure 3). Most of the VUS were missense variants (77/86, 90%), with the rest including two frameshift variants, three splice variants, three in-frame indels, and one loss-of-initiation-codon variant. One missense variant from LOVD—c.1085C>A p.(Ala362Asp)—was classified as likely benign because of the BS1 criterion and the absence of any evidence for pathogenicity. A complete list of variants and the evidence for their classification are presented in Table S1.

**Figure 4:**
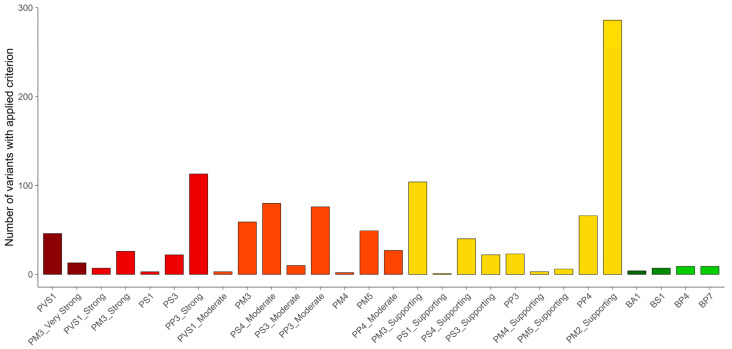
Summary of variant interpretation criteria applied to the 306 *GCDH* variants in LOVD.

As shown in Figure 4, the PM2 criterion (at a supporting level) was applied to most of the variants (286/306), reflecting the rarity of the disease and disease-associated alleles in the general population. In addition to the most frequent pathogenic variant p.(Arg402Trp), further seven variants—p.(Ala421Val), p.(Ala421Thr), p.(Val400Met), p.(Ala293Thr), p.(Arg227Pro), p.(Arg94Gln), and c.1244-2A>C p.(?)—had allele frequencies above the PM2 and below the BS1 thresholds. Nevertheless, their classification as (likely) pathogenic remained, even when the PM2 criterion was disregarded.

Overall, 69% (212/306) of *GCDH* variants had in silico computational evidence of pathogenicity (PP3) at different levels, and 18% (54/306) had graded evidence of pathogenicity from functional studies (PS3) (Figure 4). Interestingly, the criterion PP3_Strong was used more frequently than PP3_Moderate or PP3 criteria (113 variants vs. 76 and 23, respectively) without any obvious hotspots of these variants in the gene.

Because the variants were not all linked to more than one individual in LOVD, the PS4 criterion was applied to 120/306 variants at different strength levels (Figure 4). The PS4_Moderate criterion was used for most variants (80/120) followed by the PS4_Supporting criterion (40/120). Only 18 variants have been reported in ≥10 unrelated affected individuals.

The strength of genotype-related PM3 and PP4 criteria was set as dependent on confirmation of variant phasing, and this information was only available for a minority of individuals in LOVD (161/842). Therefore, the PP4 criterion was less frequently applied at an upgraded level of evidence (66 times PP4 vs. 27 times PP4_Moderate) (Figure 4). The same was true for the PM3 criterion, which could not be used for 72 variants, although the patient data were available in LOVD. PM3 was applied at a supporting level of evidence to 104 variants, 58 of which were reported in a single unrelated affected individual or in a single family. Nevertheless, as the proband count contributes to the strength of PM3, a moderate, strong, and very strong level of evidence was used for 26, 6, and 4 variants, respectively, even without information on confirmed phasing.

Of 86 variants classified as VUS, 11 were submitted to LOVD without any information on the corresponding individuals. Of the 75 remaining VUS, 47 were heterozygous, 29 were homozygous, and one was reported in both states. Sixty-nine VUS were identified in one unrelated affected individual or in a single family. For seven variants, PP4 was used because residual activity was measured in the patient fibroblasts. Phasing of variants was reported for six homozygous and two heterozygous VUS identified in one individual or single family each, enabling the use of PM3_Supporting and PM3 criteria, respectively. The PM3_Supporting criterion was also applied to an additional 25 variants, for which in trans occurrence with a pathogenic variant was assumed.

Variants with VUS classification were further split according to the VUS temperature scale ranging from “ice-cold” (low level of supporting evidence for pathogenicity) to “hot” (high level of supporting evidence for pathogenicity) to highlight VUS that are most likely to be pathogenic variants if additional proof of pathogenicity can be obtained. This scale is suggested by ACGS Best Practice Guidelines 2020 and reflects different scores of VUS calculated by utilizing the Bayesian point system [18,24,25]. As shown in Table 2, most VUS (43/75, 57%) linked to at least one individual in LOVD are grouped into “hot” or “warm” categories. In contrast, approximately two-thirds of VUS without information on individuals (7/11, 64%) are in the middle or at the “cooler” end of the scale.

Seven of 32 variants without patient data were classified as likely pathogenic (a) three times on the basis of alteration type (frameshift) by meeting the PM2_Supporting criterion, (b) three times because of existing functional analyses, and (c) once because of the combination of PP3_Strong, PM2_Supporting, and PM5 criteria. Interestingly, two variants—p.(Met100Ile) and p.(Gln251Arg)—were submitted to LOVD as likely benign, but were reclassified as VUS because of their allelic rareness in population databases and because sufficient evidence of their benign status was not provided.

### 3.3. Geographic Distribution of GCDH Variants

#### 3.3.1. Global Distribution of GA-1 Patients in LOVD

**Figure 5:**
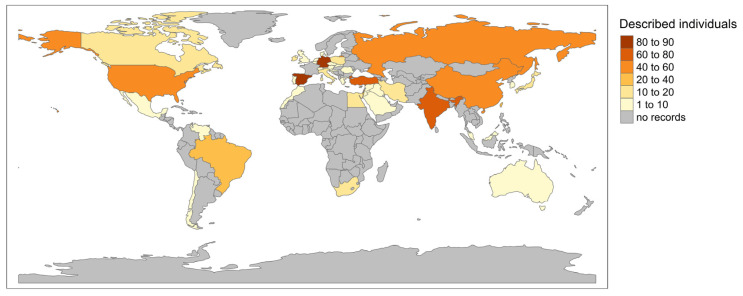
Geographic distribution of GA-1 individuals in LOVD. For exact numbers of described individuals and percentage distribution, see Table S2.

Entries of GA-1 individuals in the LOVD database with available genotype and geographic information were extracted for 759 individuals (1504 alleles). The geographic distribution of the records is shown in Figure 5. The number of reported individuals differed greatly between countries; for instance, the three countries with most records were Spain (90 individuals; 12% of records), Germany (85 individuals; 11% of records), and Turkey (76 individuals; 10% of records), while in eight countries, only one individual was described (each representing 0.13% of all records). In 76% of countries, fewer than 20 individuals were recorded, and in many countries, no individuals were reported at all.

#### 3.3.2. Variant Distribution in Specific Regions

**Figure 6:**
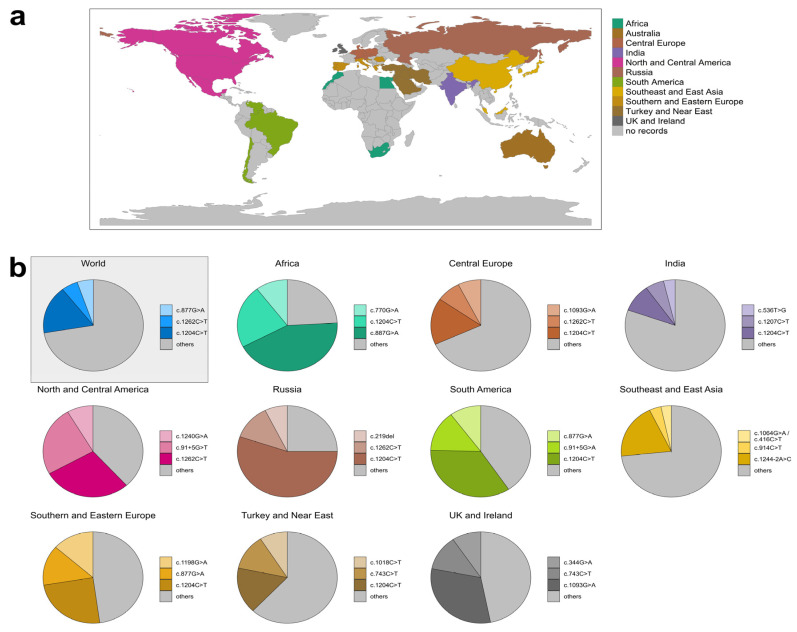
Distribution of the three most abundant variants in 11 regions. (**a**) Countries that were combined into 11 geographic regions. (**b**) Distribution of the three most abundant variants worldwide and in 10 regions. Australia is not shown because too few data were available. In Southeast and East Asia, variants c.1064G>A and c.416C>T occurred with equal frequency.

To examine the geographic distribution of variants, data derived from related geographic countries were combined as described above (Figure 6a). Within these defined regions, all available variants were analyzed according to their occurrence and the three most abundant variants were extracted (Figure 6b and Table S3). In Australia, only 12 variants have been described, with the most frequent variant being described a maximum of twice. Thus, data were considered insufficient for more elaborate distribution analysis. For Southeast and East Asia, two variants—c.1064G>A p.(Arg355His) and c.416C>T p.(Ser139Leu)—were equally common and thus both considered as the third-most frequent variant in this region.

Worldwide, 1504 alleles and 265 different variants were described. The most common variant (17.09% of all described variants) was c.1204C>T p.(Arg402Trp) (257 recorded alleles), followed by c.1262C>T p.(Ala421Val) (80 recorded alleles; allele frequency 5.32%), and c.877G>A p.(Ala293Thr) (79 recorded alleles; allele frequency 5.25%). Variant c.1204C>T p.(Arg402Trp) was found in all analyzed regions except Australia and was the most or second-most common variant in seven regions (Africa, Central Europe, India, Russia, Southern and Eastern Europe, and Turkey and Near East). Remarkably, variant c.1204C>T p.(Arg402Trp) accounted for more than 55% of all described variants in Russia. The second-most common variant worldwide was c.1262C>T p.(Ala421Val) and was mainly described in Central Europe, North and Central America, and Russia. The third-most common variant was c.877G>A p.(Ala293Thr), which was mainly described in South Africa, Spain, and Israel, and was the third most common variant in South America.

The most common variants of Southeast and East Asia were rather exclusive to this region, with c.1244-2A>C p.(?) being described 41 times in Southeast and East Asia and only once outside this region in Australia. The second-most common variant of this region was c.914C>T p.(Ser305Leu) and the third-most common were c.1064G>A p.(Arg355His) and c.416C>T p.(Ser139Leu). Of these respective variants, roughly 57%, 70%, and 70% worldwide were recorded in Southeast and East Asia.

Interestingly, the described variants seldom occurred, with ≈63% of worldwide variants only being described once or twice (166/265 variants) and only ≈10% being described more than 10 times (27/265 variants) (Table S4).

From a total of 759 genotypes, the three most prevalent genotypes worldwide were c.[1204C>T]; [1204C>T] p.[Arg402Trp]; [Arg402Trp] (77/759 individuals; ≈10%), c.[1262C>T]; [1262C>T] p.[Ala421Val]; [Ala421Val] (25/759 individuals; ≈3%), and c.[877G>A]; [877G>A] p.[Ala293Thr]; [Ala293Thr] (25/759 individuals; ≈3%). Of the 10 most common genotypes, only one was heterozygous: c.[1204C>T]; [877G>A] p.[Arg402Trp]; [Ala421Thr] (12/759 individuals; ≈1.6%). Remarkably, 72.3% of all genotypes were detected in one individual only (Table S5).

## 4. Discussion

A considerable proportion of *GCDH* variants in LOVD (28%, 86/306) were classified as VUS after applying ACMG/AMP variant interpretation criteria adapted and specified for *GCDH*. For 83 variants, this classification conflicted with the previous interpretation as (likely) pathogenic in the literature. Of note, the overwhelming majority of these variants were detected in one affected individual with no further evidence of their pathogenicity. Many papers included in this study were published either before the release of ACMG/AMP guidelines for clinical variant interpretation [16] or before these guidelines became an integral part of molecular genetic diagnostics. Thus, the classification of variants has largely relied on patient’s phenotype and, to some degree, on zygosity of detected variants (homozygous or heterozygous with a co-occurrent second variant). It is important to mention that genotypes—compound heterozygous and homozygous—remain assumed if the variant phase has not been proven. Few papers stated that parental samples had been tested [38,39,40], supporting the pathogenicity of the detected variants in the index patient in line with the autosomal-recessive GA-1. PM3, which addresses this evidence of pathogenicity, is a powerful criterion because of its point-based nature. For example, if a suspicious variant is detected in trans with a pathogenic or likely pathogenic variant and the phase is confirmed, PM3 could be applied at a moderate level. If the phase is unknown, the second variant must be pathogenic and PM3 could be applied with downgraded weight only. Homozygous occurrences, which are frequently described for *GCDH* variants, as well as co-occurrences of two VUS cannot be assigned with PM3 without confirmed phasing. PM3 could be applied to variants detected in co-occurrence with a likely pathogenic variant if the phase is unknown, but with the lowest point value. Thus, for moderate evidence of pathogenicity, the variant has to be found in unconfirmed compound heterozygosity with four different likely pathogenic variants—a scenario less realistic for GA-1. In contrast, two affected compound heterozygotes with two known likely pathogenic variants and a known phase are enough to upgrade PM3 to the strong level of evidence. From 75 VUS with patient data, the phase was known only for eight variants, whereas it was just assumed for the remaining 67 VUS. Of note, 26 VUS could be reclassified as likely pathogenic using PM3 with an increased strength. This clarifies how a straightforward but clear statement of confirmed phasing can enable upgrading of variant pathogenicity at minimal cost.

Almost all *GCDH* variants in LOVD were extracted from the literature, and published phenotypes of corresponding individuals varied greatly, depending on the focus of individual studies. Thus, the choice of an appropriate GA-1-specific phenotype as evidence for the PP4 criterion was challenging due to heterogeneous and inconsistent description of clinical symptoms and biochemical findings. Furthermore, several asymptomatic individuals with biochemical phenotypes suspicious for GA-1 have been reported so far [41,42,43], making biochemical features alone less appropriate for PP4. To overcome these limitations, PP4 was applied only on (both) variants of genotypes that were identified in individuals with decreased or absent GCDH activity in their fibroblasts or lymphoblasts. Impaired enzyme activity in vivo represents a phenotypic feature of an individual and strengthens the specificity of a single genetic etiology, even though the data support the pathogenicity of the genotype rather than that of the variants. In this study, PP4 was increased in strength if biallelic occurrence of *GCDH* variants was confirmed by parental testing, supporting the evidence that both variants contribute to the phenotype. However, assessment of GCDH activity is not a standard confirmation of diagnosis, and is not usually available in diagnostic laboratories. This issue is also reflected by the low number of individuals with measured enzyme activity in LOVD (161/842). Future approaches should address further disease-specific phenotypes of GA-1 or a valuable combination of them, which is adequate for the use of PP4. For this purpose, the clinical characteristics should be reported in a standardized way. The use of Human Phenotype Ontology (HPO, https://hpo.jax.org/app/; accessed on 10 October 2023) might be the most beneficial way.

Very few or no *GCDH* variants in affected individuals have been reported in population databases. In LOVD, 286/306 *GCDH* variants, including all variants classified as VUS, have allele frequencies below the calculated cutoff for PM2. This criterion was used at a supporting level of evidence, as recommended by the SVI working group [37]. This downgrading of the PM2 weight may cause many variants previously classified as likely pathogenic to become VUS; however, other criteria were also used with upgraded weighting. For example, PP3 was applied at a moderate or even strong level of evidence on 41/86 VUS in LOVD.

As no specific structural domains of *GCDH* have been significantly associated with missense constraints in DECIPHER, the PM1 criterion was not applied to the evaluated variants in LOVD. However, according to ACGS 2020 guidelines, in silico protein modeling data may be considered when assessing variant pathogenicity through the PM1 criterion. The crystal structure of human GCDH complexed with alternate substrate 4-nitrobutyryl-CoA has been determined and is available in the Research Collaboratory for Structural Bioinformatics Protein Data Bank (PDB) (PDB code: 1SIR) [32]. Several studies used this model to investigate potential effects of *GCDH* missense variants on protein folding and stability, encompassing novel variants identified in a patient as well as large sets of already known variants and genotypes [12,28,29,44,45]. A consistent assessment of these results is challenging because different approaches were used. In the last few years, numerous innovative structure-based algorithms for variant prediction have been developed, including those based on deep learning [46]. Thus, efforts should be made to evaluate the validity and utility of one or a combination of different in silico protein structural methods when classifying *GCDH* variants, as has already been done for other genes [47,48].

This study highlights the importance of systematic and thorough collection of data concerning GA-1-associated genotypes and phenotypes, including information on genetic testing of relatives and research data. The LOVD dataset has provided a comprehensive overview of *GCDH* variants, enabling a disease-specific framework for variant classification. As this dataset grows continuously, the adjustment of variant interpretation criteria to *GCDH* could be optimized, allowing renewed variant curation.

The LOVD dataset can be used to analyze the spatial distribution of *GCDH* variants, which was performed here. The amount of available data varied considerably between countries. This may partly be explained by differences in NBS between higher-income countries such as Germany (with early diagnosis of many diseases) and low- and middle-income countries such as most African countries (with no or limited screening) [49,50]. Also, the availability of molecular genetic testing differs considerably within different countries [51]. However, data on *GCDH* variants and their phenotypes were also unavailable in countries with well-established NBS for GA-1 and with widespread molecular genetic testing, such as Norway, Sweden, and Denmark. This has practical implications since the classification of variants in diagnostic laboratories is based on information on previously described variants. Most *GCDH* variants are missense changes and are challenging to classify; therefore, the limited data on pathogenic *GCDH* variants in some countries may contribute to their classification as VUS. This is particularly interesting as the geographic distribution of variants differed considerably, e.g., the variant c.1018C>T p.(Leu340Phe) was one of the most common variants in Turkey, but has not been reported in other regions yet. It can be assumed that the variant patterns in several regions with no or limited data on *GCDH* variants also differ from those found in countries with more data on *GCDH* variants, such as in Central Europe. GA-1 patients from Asian and African countries are particularly proportionally underrepresented in the literature.

The reasons for the spatial heterogeneity of *GCDH* variants are largely unknown. Most variants in Southeast and East Asia are exclusive to this region. This is consistent with other studies showing a different pattern of pathogenic variants for several rare disorders in Asia compared with Europe or the United States [52,53]. Selective pressure for heterozygous carriers of *GCDH* variants (as described in carriers of cystic fibrosis who have a selective advantage against cholera and other diarrheal disease) is rather unlikely in GA-1 [54]. For GA-1, several genetic isolates sharing the same variant have been reported with higher prevalence in some populations compared with the worldwide prevalence. For instance, in the aboriginal population of Island Lake, Manitoba and northwestern Ontario, GA-1 has a higher prevalence and c.91+5G>T p.(?) was the only pathogenic allele identified among affected individuals [55,56,57,58,59]. The geographic distribution of *GCDH* variants is also relevant for further studies, e.g., on the correlation of genotype and clinical phenotype. Early initiation of metabolic treatment dramatically improves the clinical outcome of patients with GA-1 [5,6,7,8]. Not only the spectrum of *GCDH* variants but also the availability of diagnostic tools (e.g., NBS) and effective treatment varies considerably between different geographic regions. Studies must consider this to avoid potential bias in their results. The advantage of LOVD is that several additional information and potential confounders for the interpretation of data (such as start of treatment) can be added and considered in further research and for diagnostic and scientific purposes.

## Figures and Tables

**Table 2:** Classifications of all unique variants in *GCDH* from LOVD, grouped by variants with and without available patient data. VUS were subclassified according to ACGS Best Practice Guidelines 2020 [18].

Classification	Variants with Patient Data	Variants without Patient Data
Pathogenic	93	0
Likely pathogenic	105	7
VUS (hot)	22	3
VUS (warm)	21	1
VUS (tepid)	20	5
VUS (cool)	8	1
VUS (cold)	4	1
VUS (ice-cold)	0	0
Likely benign	0	6
Benign	1	8
Total	274	32

## Data Availability

Data on all newly curated *GCDH* variants and patients’ data are accessible on LOVD (https://www.lovd.nl).

## Supplementary Materials

The following supporting information can be downloaded at https://www.mdpi.com/article/10.3390/genes14122218/s1. Table S1: Assessment for variant classification; Table S2: Geographic distribution of GA-1 individuals in LOVD; Table S3: The three most common variants worldwide and per region; Table S4: Variant frequency worldwide; Table S5: Genotype prevalence worldwide.
